# Light-induced propulsion of a giant liposome driven by peptide nanofibre growth

**DOI:** 10.1038/s41598-018-24675-7

**Published:** 2018-04-19

**Authors:** Hiroshi Inaba, Akihito Uemura, Kazushi Morishita, Taiki Kohiki, Akira Shigenaga, Akira Otaka, Kazunori Matsuura

**Affiliations:** 1 Department of Chemistry and Biotechnology, Graduate School of Engineering, Tottori University, 4-101 Koyama-Minami, Tottori, 680-8552 Japan; 2 Institute of Biomedical Sciences and Graduate School of Pharmaceutical Sciences, Tokushima University, Shomachi, Tokushima, 770-8505 Japan

## Abstract

Light-driven nano/micromotors are attracting much attention, not only as molecular devices but also as components of bioinspired robots. In nature, several pathogens such as *Listeria* use actin polymerisation machinery for their propulsion. Despite the development of various motors, it remains challenging to mimic natural systems to create artificial motors propelled by fibre formation. Herein, we report the propulsion of giant liposomes driven by light-induced peptide nanofibre growth on their surface. Peptide-DNA conjugates connected by a photocleavage unit were asymmetrically introduced onto phase-separated giant liposomes. Ultraviolet (UV) light irradiation cleaved the conjugates and released peptide units, which self-assembled into nanofibres, driving the translational movement of the liposomes. The velocity of the liposomes reflected the rates of the photocleavage reaction and subsequent fibre formation of the peptide-DNA conjugates. These results showed that chemical design of the light-induced peptide nanofibre formation is a useful approach to fabricating bioinspired motors with controllable motility.

## Introduction

Molecular robotics is a recently emerged concept for the construction of bioinspired robots composed of nano- and micro-scale devices, such as sensors, logic circuits and actuators^1–3^. By using these components, a molecular robot responds to its surrounding environment and makes decisions autonomously. For instance, Nomura *et al*. recently developed an amoeba-like molecular robot consisting of a body (liposome), motor (kinesin, microtubules) and clutch (DNA)^3^. When the kinesin was attached to a liposomal inner membrane in response to a signal molecule composed of complementary DNA, the robot exhibited a continuous shape change caused by microtubules gliding on the membrane. Some of the important components of molecular robots are self-propelled nano- and micro-scale motors, which are propelled by converting external energy to mechanical motion^4–11^. Compared with the above kinesin-microtubule system, a simple strategy for propelling a motor is the induction of an energy gradient between its two sides^4–11^. One of the major driving forces of the propulsion is the Marangoni effect, caused by a surface tension gradient. This gradient propels the motor from its low-surface-tension side to its high-surface-tension side^4,12,13^. Based on these design principles, various nano/microparticles with asymmetric catalytic sites have been developed to convert chemical energy to mechanical motion^14–22^. In the presence of substrates, a catalytic motor generates an asymmetric distribution of products, which causes diffusiophoresis^14–20^, thermophoresis^21^ and pH phoresis^22^. In addition to catalytic motors, light-driven motors have been recently developed because light is an attractive energy source for modulating the movement of motors by switching the on/off timing and orientation of light^23–41^. For instance, Ren *et al*. reported the construction of TiO_2_, BiOI and carbon WO_3_-based Janus micromotors, which were propelled by photocatalytic electrophoresis^23,24^ or diffusiophoresis^25^. A photothermal effect has been utilised to propel a gold-based motor by the light-induced formation of a thermal gradient across the motor, resulting in thermophoresis^26–28^. Sugawara *et al*. constructed oil droplets in a metal-free system. This was done by decomposing photolabile 2-nitrobenzyl oleate by UV light irradiation to generate a partial oleic acid distribution on the surface, resulting in the propulsion of oil droplets by the Marangoni effect^29^.

To design new light-driven motors, we focused on the self-propelled motility of several bacterial pathogens driven by nanofibre growth. Actin polymerisation on the surface of *Listeria monocytogenes* and *Shigella flexneri* forms a local network of actin filaments called an ‘actin comet tail’, which provides a powerful force to propel the bacteria through host cell cytoplasm^42^. The moving mechanisms of *Listeria* have been investigated by the activation of actin polymerisation on the surface of liposomes and polystyrene microspheres, which has been achieved by coating them with the *Listeria* transmembrane protein ActA^43,44^. Thus, light-induced nanofibre growth can be used as a new propulsion force for light-driven motors. Peptides are promising building blocks for this purpose because (1) light-responsive amino acids can be introduced in peptides and (2) nanofibre-forming peptide sequences can be designed. Amyloidogenic, β-sheet and β-hairpin-forming peptides have been conjugated with light-responsive moieties such as the azobenzene, nitrobenzyl and spiropyran groups for light-induced fibre formation^45–48^.

**Figure 1:**
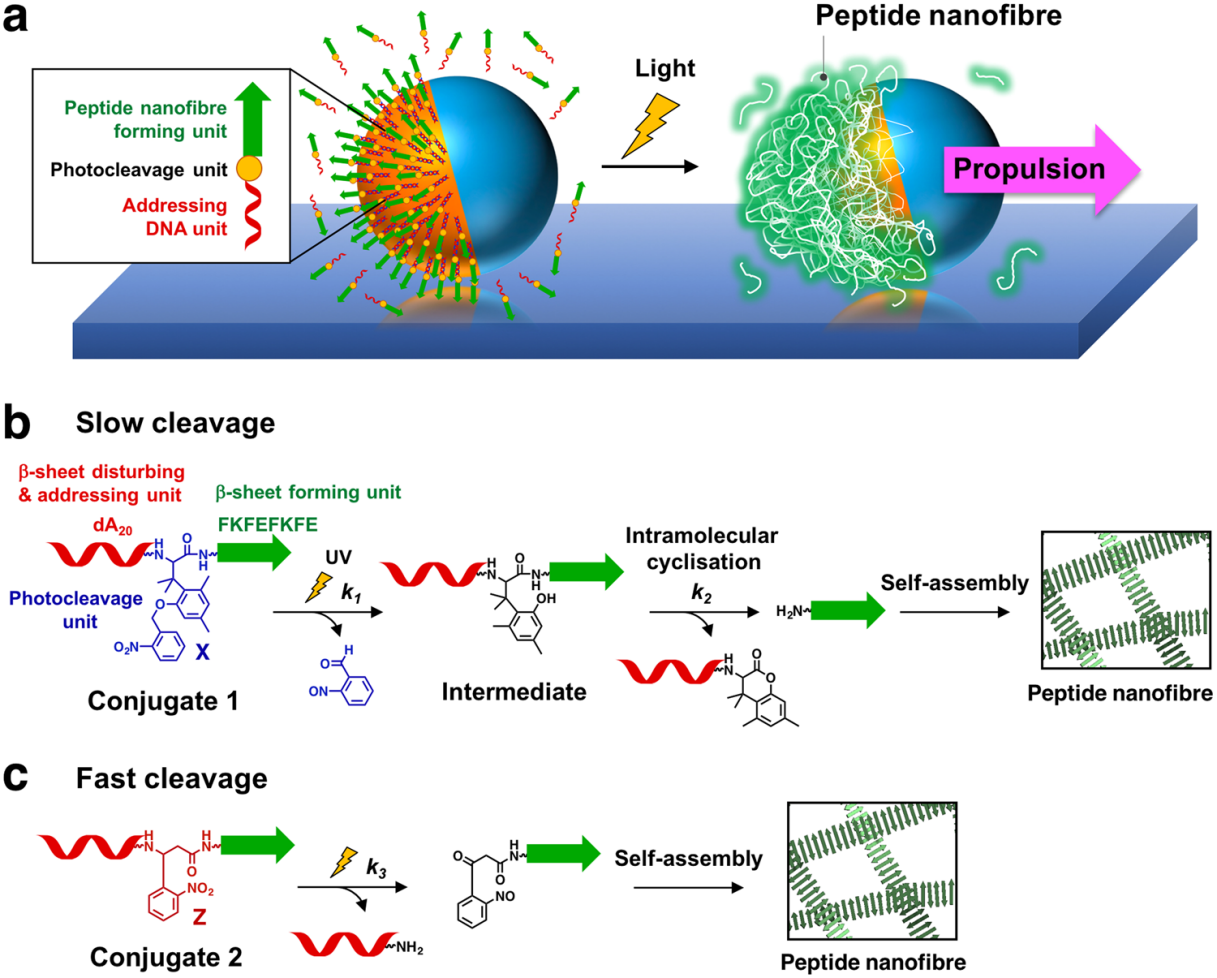
Design of a light-driven motor using photo-cleavable peptide-DNA conjugates. (**a**) Light-induced propulsion of a phase-separated liposome driven by local peptide nanofibre growth. The photocleavage reaction and peptide nanofibre formation of (**b**) conjugate **1** and (**c**) conjugate **2**.

Herein, we constructed light-driven motors that were propelled by light-induced fibre formation on their surfaces (Fig. 1a). A peptide nanofibre-forming unit with a photocleavage unit was asymmetrically conjugated on the surface of giant liposomes by DNA hybridisation of the addressing unit. By light irradiation, the peptide bonds were cleaved and nanofibre-forming peptides were released around the liposome. The released peptides subsequently self-assembled to form nanofibres, which could induce propulsion of the liposome by a mechanism similar to actin polymerisation-induced motility. We previously reported a light-induced peptide nanofibre growth system using conjugate **1**, consisting of β-sheet-forming FKFEFKFE peptide and addressing single-stranded DNA (dA_20_), which are linked by a photo-cleavable amino acid X (Fig. 1b)^49^. Ultraviolet (UV) irradiation to conjugate **1** releases free FKFEFKFE peptide, which self-assembles to form peptide nanofibre. In this work, we newly synthesised conjugate **2** to increase the cleavage rates of the peptide bond compared with conjugate **1** (Fig. 1c). Conjugates **1** and **2** were introduced onto the surfaces of the liposomes, and the light-induced translational movements of the liposomes were analysed.

## Results

### Kinetics of light-induced fibre formation of conjugates 1 and 2

Conjugate **2** was designed by replacing the photoresponsive amino acid X of conjugate **1** with amino acid Z for a faster photocleavage reaction. Upon light irradiation of conjugate **1**, the unprotected phenolic intermediate was generated and then cleaved into dA_20_ and peptide moieties by intramolecular cyclisation (Fig. 1b). The intramolecular cyclisation was expected to be slow, especially when the sterically hindered residue was introduced to the position adjacent to X^50^. By contrast, Z could be cleaved by light without the intramolecular cyclisation that took place in the case of X (Fig. 1c). 9-Fluorenylmethyloxycarbonyl (Fmoc) derivatives of Z are commercially available and have been used for light-induced self-assembly^45^, conformational change^51^, binding to the epitopes of peptides^52^, and control of DNA binding^53^. Conjugates **1** and **2** were synthesised by our reported procedure (see Methods and Supplementary Scheme 1).

The photocleavage reactions of conjugates **1** and **2** were monitored using reverse phase high-performance liquid chromatography (RP-HPLC). The processing products were characterised by matrix-assisted laser desorption/ionisation time-of-flight mass spectrometry (MALDI-TOF-MS) (refer to Supplementary Information). Upon UV irradiation (365 nm, 4 W cm^−2^) into conjugate **1**, the phenolic intermediate was immediately generated. The reaction showed a first-order rate dependence, and the kinetic constant *k*_1_ and half-life t_1/2_ were determined as 0.42 sec^−1^ and 1.7 sec, respectively (Supplementary Fig. 3). By UV irradiation into conjugate **1** for 30 sec and subsequent incubation at 25 °C for 0–130 min, the intermediate was gradually cleaved. In this cleavage reaction, *k*_2_ and t_1/2_ were determined as 0.17 × 10^−3^ sec^−1^ and 4380 sec, respectively (Supplementary Fig. 5). Thus, the intramolecular cyclisation of the phenolic intermediate was extremely slow, as expected. By contrast, UV irradiation of conjugate **2** caused immediate cleavage with a first-order rate dependence. In the reaction, *k*_3_ and t_1/2_ were determined as 0.05 sec^−1^ and 14 sec, respectively (Supplementary Fig. 7). These results showed that the cleavage reaction of conjugate **2** was obviously faster than that of conjugate **1**.

The increment of the turbidity (optical density at 400 nm) of conjugates **1** and **2** caused by to light-induced fibre formation was monitored (refer to Supplementary Information). Upon UV irradiation for 3 min, the turbidity of conjugate **2** was dramatically increased because of the fibre formation, whereas conjugate **1** showed only a slight change (Supplementary Fig. 9). This result indicated that conjugate **2** was cleaved by UV irradiation, resulting in the release of the peptide moiety to form the nanofibres immediately. However, the cleavage reaction of conjugate **1** was too slow to produce nanofibres from 3 min of UV irradiation. The light-induced fibre formation of conjugate **2** was observed by transmission electron microscopy (TEM) (Supplementary Fig. 10). These results indicated that the rates of fibre formation could be modulated by changing the photocleavage moiety.

### Light-induced translational movement of giant liposomes

**Figure 2:**
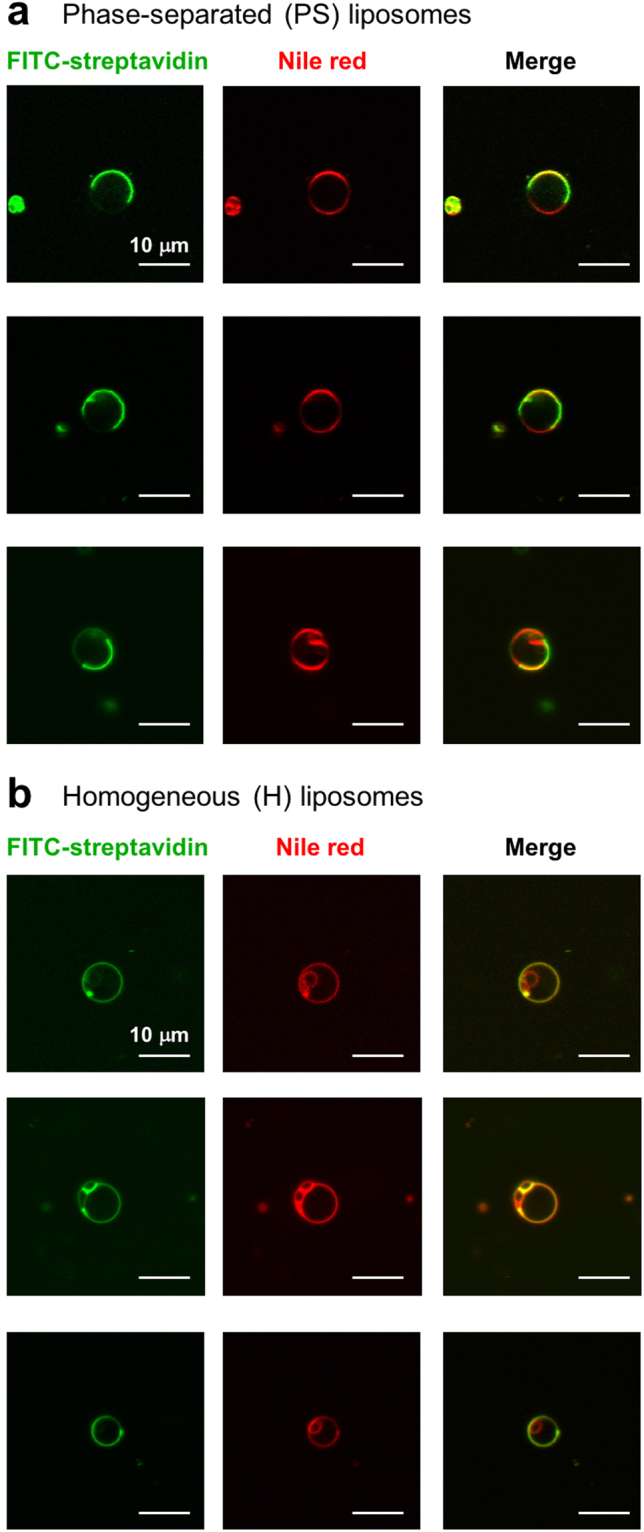
Phase separation and homogeneity of liposomes. CLSM images of (**a**) phase-separated (PS) and (**b**) homogeneous (H) liposomes stained with FITC-streptavidin (green) and Nile red (red).

Because the asymmetric distribution of actin filaments is important for the propulsion of the actin-based motors^43,44^, phase-separated giant liposomes (PS-liposomes) were prepared for the asymmetric modification of conjugates **1** and **2** (Supplementary Fig. 11). Liposomes composed of a saturated phospholipid 1,2-dipalmitoyl-*sn*-glycero-3-phosphocholine (DPPC), an unsaturated lipid 1,2-dioleoyl-*sn*-glycero-3-phosphocholine (DOPC) and cholesterol (Chol) are known to phase-separate into coexisting liquid phases by optimising the mixing ratio of the three components^54,55^. DPPC and Chol form a liquid-ordered (L_o_) domain, and DOPC forms a liquid-disordered (L_d_) domain in a single liposome. 1,2-Dioleoyl-*sn*-glycero-3-phosphoethanolamine-*N*-(cap biotinyl) (sodium salt) (DOPE-biotin) was used for the localisation of biotin on the L_d_ domains of PS-liposomes. Using a natural swelling method, we prepared typically sized 10 to 30-μm PS-liposomes consisting of DPPC/DOPC/DOPE-biotin/Chol with a molar ratio of 5:4:1:4.5. The **1**- and **2**-modified PS-liposomes (**1**-PS and **2**-PS) were prepared by the selective conjugation of **1** and **2** on the L_d_ domain of the PS-liposomes, which was achieved by the sequential conjugation of **1** and **2** with dT_20_-biotin molecules (dA_20_-dT_20_ complementary hybridisation), streptavidin (streptavidin-biotin interaction) and the DOPE-biotin of PS-liposomes (streptavidin-biotin interaction) (Supplementary Fig. 11). To facilitate the fibre formation under light irradiation, the excess amounts of conjugates **1** and **2** (1.5 equivalent to dT_20_-biotin) were used because the amount of the released peptide moiety is important for fibre formation. For the comparison with PS-liposomes, we also prepared homogeneous liposomes (H-liposomes) consisting of DOPC/DOPE-biotin/Chol with a molar ratio of 9:1:4.5. The phase separation and homogeneity of the PS- and H-liposomes, respectively, were confirmed by the localisation of FITC-labelled streptavidin to the DOPE-biotin on the liposomes (Fig. 2). The localised fluorescence of FITC-streptavidin on PS-liposomes indicates the binding of FITC-streptavidin to biotin on L_d_ domain of PS-liposomes (Fig. 2a). In contrast, the homogeneous fluorescence of FITC-streptavidin on H-liposomes indicates the binding of FITC-streptavidin to homogeneously-distributed biotin on H-liposomes (Fig. 2b). By light irradiation of **1**-PS, the formation of peptide nanofibres surrounding the liposomes was observed by TEM (Supplementary Fig. 12).

**Figure 3:**
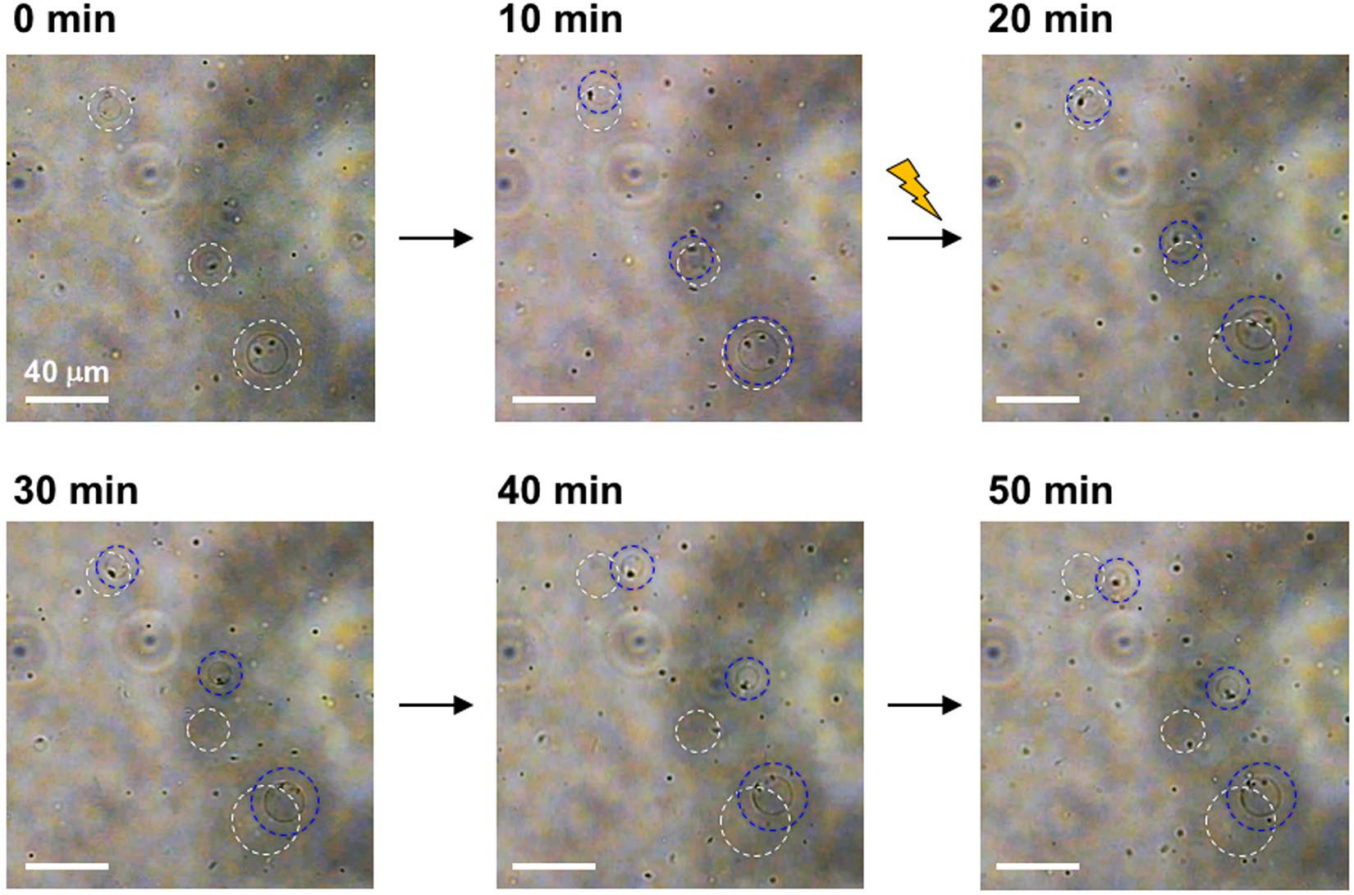
Snapshots of 2-PS with UV light irradiation. White and blue circles represent the initial and final positions of the liposomes, respectively. The UV light irradiation lasted for 10–20 min of recording.

**Figure 4:**
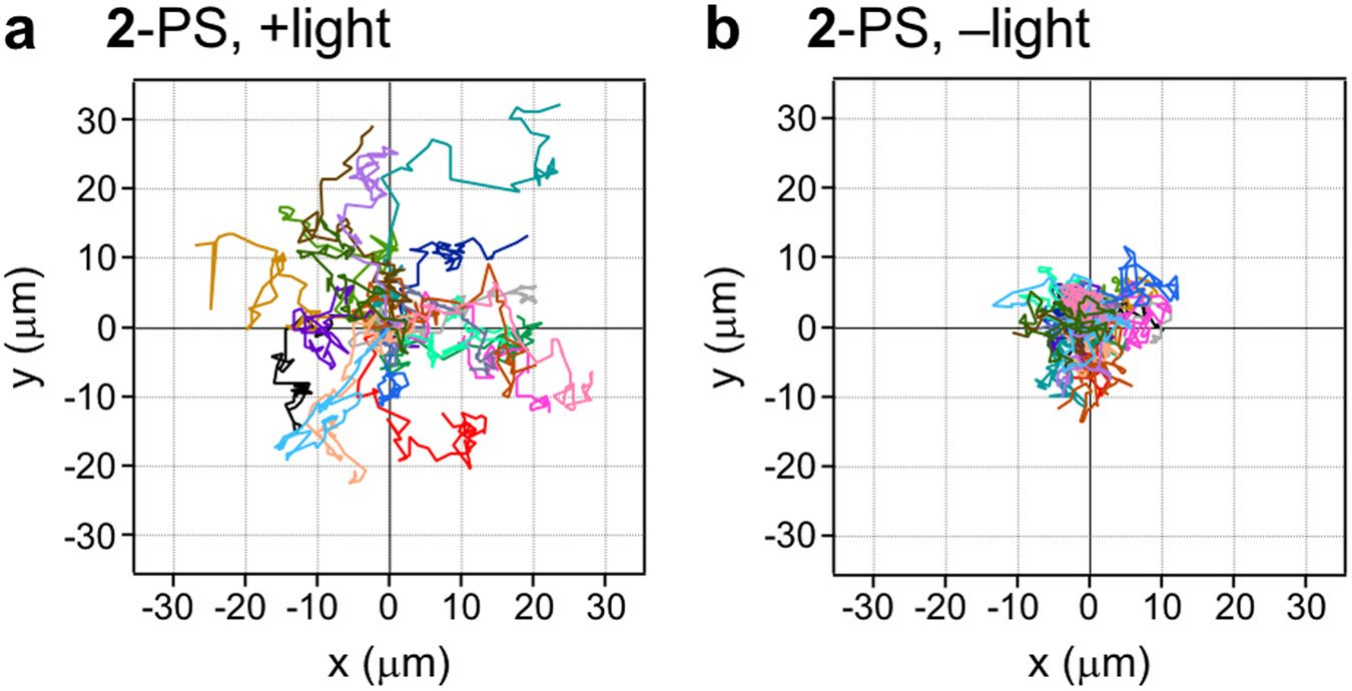
Tracking trajectories of 2-PS. The trajectories were recorded during 60 min (**a**) with and (**b**) without UV light. Irradiation lasted for 10–20 min of recording for (**a**). Each colour represents a different liposome (*N* = 20).

**Figure 5:**
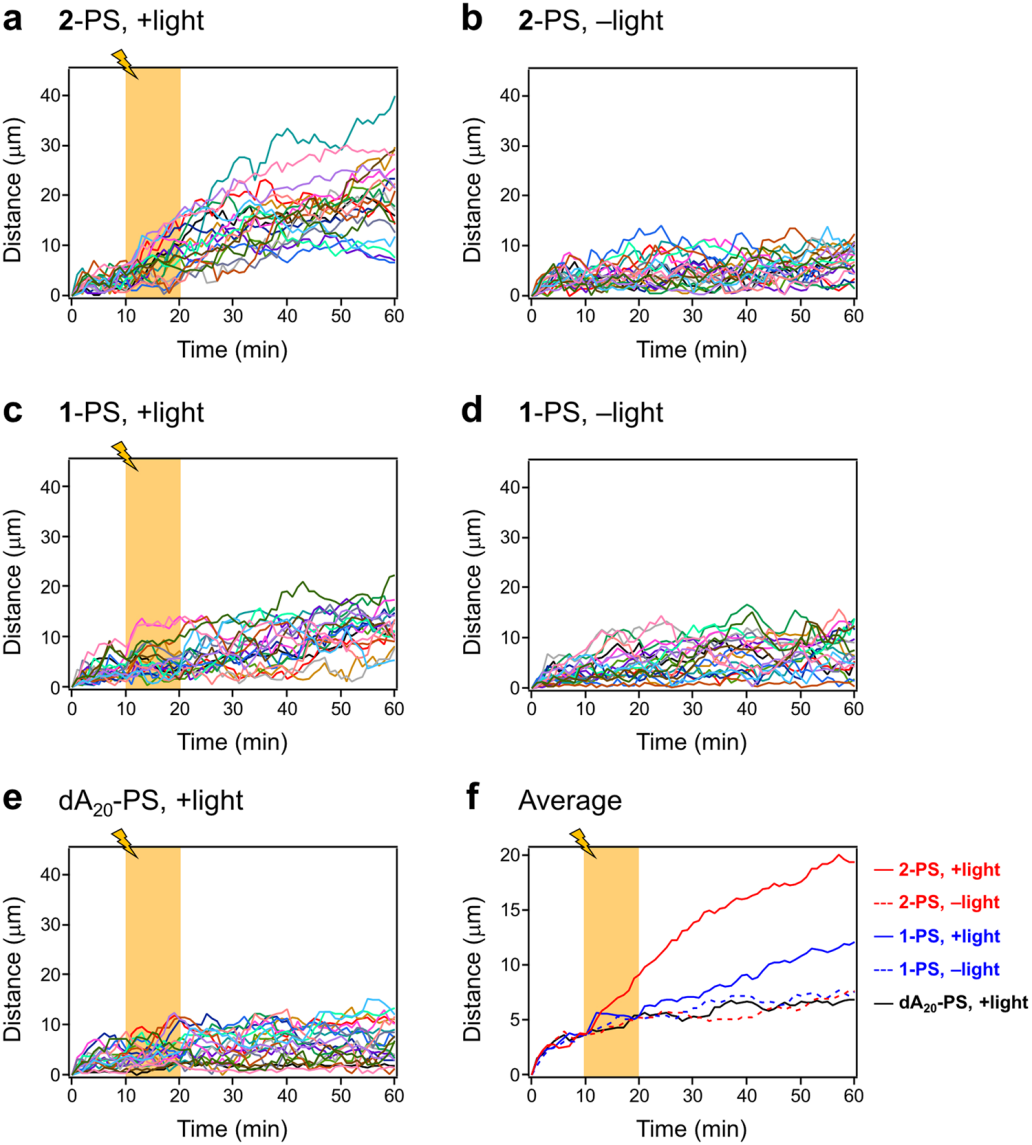
Time dependence of the distance of the PS-liposomes from the initial position. The distance was analysed from the trajectory tracking: (**a**,**b**) **2**-PS; (**c**,**d**) **1**-PS; (**e**) dA_20_-PS and (**f**) average of (**a**–**e**) under UV light irradiation (orange zones) and non-irradiation (white zones). In (**a**–**e**), each colour represents a different liposome (*N* = 20).

The light-induced translational movements of **1**-PS and **2**-PS were monitored by optical microscopy. As a control, we prepared PS-liposomes, which were modified with a dA_20_ without a peptide moiety (dA_20_-PS). In the microscopic observation, we selected similar size of liposomes (13–16 μm) for comparison (Supplementary Table 1). In the light-irradiation experiments, the movement of liposomes was monitored under darkness for 10 min, followed by UV light irradiation for 10 min, then again under darkness for 40 min with the settings shown in Supplementary Fig. 13. Snapshots of **2**-PS show the enhanced propulsion of **2**-PS after light irradiation (Fig. 3, captured from Supplementary Video 1). The trajectories of **1**-PS, **2**-PS and dA_20_-PS are plotted in Fig. 4 and Supplementary Fig. 14. The movement of **2**-PS exhibits a typical random walk caused by Brownian motion (Fig. 4). The moving distance of **2**-PS was much longer under light irradiation (Fig. 4a) than under darkness (Fig. 4b), suggesting the enhanced diffusion of **2**-PS by light-induced fibre formation. The distances of **1**-PS, **2**-PS and dA_20_-PS from their initial positions were calculated from the trajectories (Fig. 5). The light irradiation promptly induced the translational movement of **2**-PS (Fig. 5a), whereas no specific movement was observed under darkness (Fig. 5b). The effect of light irradiation on **1**-PS seemed to be less than that of **2**-PS (Fig. 5c,d). The movement of dA_20_-PS was not changed by light irradiation (Fig. 5e), indicating the requirement of conjugates **1** and **2** for light-induced translational movement. The comparison of **1**-PS, **2**-PS and dA_20_-PS indicated that the local heating by the light irradiation was not the reason for the enhanced diffusion of **2**-PS because **1**-PS and dA_20_-PS showed no enhanced diffusion during the light irradiation (Fig. 5c,e). Comparison of the averages of the moving distances clearly showed that light irradiation immediately induced the translational movement of **2**-PS, whereas the response of **1**-PS to the light was slow (Fig. 5f). This difference reflected the fast fibre formation of conjugate **2** compared with conjugate **1** under light irradiation (Supplementary Fig. 9), indicating the importance of the nanofibre growth for propulsion.

## Discussion

Kinetics of photocleavage reaction of conjugate **1** and **2** were correlated with kinetics of fibre formation and the movement of **1**- and **2**-conjugated liposomes. Although the photolysis of **1** was fast (t_1/2_ = 1.7 sec), the intramolecular cyclisation of the intermediate to generate the free peptide was slow (t_1/2_ = 4380 sec) (Supplementary Figures 3 and 5). The overall slow cleavage of **1** induced slow fibre formation (Supplementary Figure 9), driving slight movement of **1**-PS (Fig. 5c). In contrast, light irradiation to conjugate **2** readily generated the peptide moiety (t_1/2_ = 14 sec) (Supplementary Figure 7) to induce the fast fibre formation (Supplementary Figure 9). The fast fibre formation was efficient to induce prompt movement of **2**-PS upon light irradiation (Fig. 5a). Thus, the chemical design of the photocleavage moiety is an efficient strategy to modulate the light-induced movement of liposomes.

**Figure 6:**
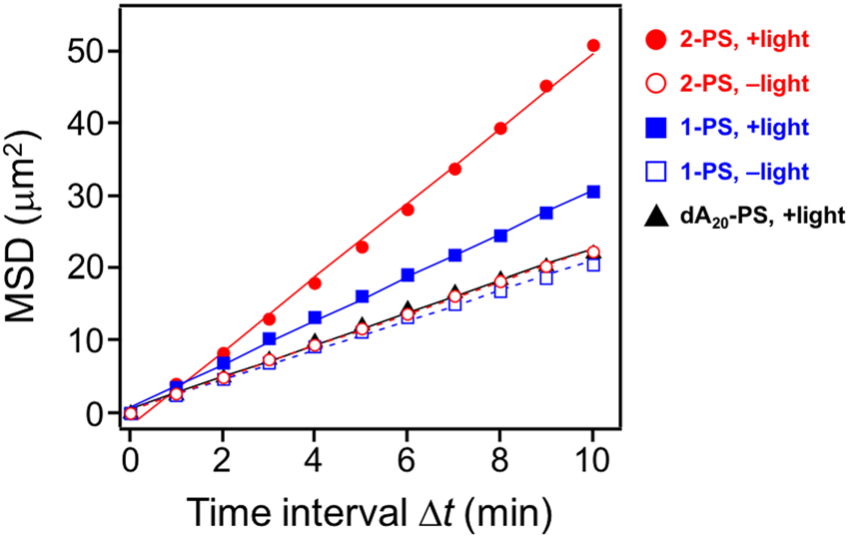
Mean square displacement (MSD) plots of PS-liposomes. Fitting plots of MSD versus time interval (Δ*t*) were analysed from the trajectory tracking of 20 liposomes during 10–60 min of measurement.

To clarify whether the movement of the liposomes was Brownian motion, the mean square displacement (MSD) of each liposome was calculated by MSD(Δ*t*) = [(**r**(*t* + Δ*t*) − **r**(*t*)]^2^, where Δ*t* is time interval and **r**(*t*) = (*x*(*t*), *y*(*t*)) is the vector position of the liposome at time *t*. The plots of MSD versus Δ*t* are shown in Fig. 6. All of the liposomes can be linearly fitted, indicating that the enhanced diffusion of **2**-PS by light-induced fibre formation was caused by non-directional Brownian motion, similar to the reported catalytic motors that generate an asymmetric distribution of products for diffusiophoresis^15,17,19,20^. The diffusion coefficient (*D*) was calculated from the fitted line, using *D* = MSD/*i*·Δ*t*, where *i* is the dimensional index, which is equal to 4 in the case of a two-dimensional analysis. The diffusion coefficients and velocities are summarised in Table 1. The diffusion coefficient of **2**-PS under darkness was 0.56 ± 0.06 μm^2^/min. With light irradiation, the value increased to 1.29 ± 0.18 μm^2^/min. In the case of **1**-PS, the diffusion coefficient was slightly increased by light irradiation (0.78 ± 0.10 μm^2^/min) compared with that under darkness (0.55 ± 0.06 μm^2^/min). The value of dA_20_-PS with light (0.58 ± 0.07 μm^2^/min) was similar to that of **1**-PS and **2**-PS under darkness. These results indicated that light irradiation of **1**-PS and **2**-PS enhanced their diffusion, which was dependent on the rates of fibre formation. The velocity of **2**-PS had a 5-fold increased under light irradiation (0.31 ± 0.04 μm/min) compared with that under darkness (0.06 ± 0.02 μm/min). Although it is difficult to compare directly, previous motility assays reported that the velocities of *Listeria* and ActA (*Listeria* transmembrane protein)-modified liposomes propelled by actin polymerisation were 1.9 ± 0.3 and 0.8 ± 0.2 μm/min, respectively^43^. The slow movement of **2**-PS compared with the actin-based motors is thought to be caused by the lower density of FKFEFKFE nanofibre in our motors compared with that of the actin filaments used by *Listeria*, as the high density of actin filaments has corresponded to a large force generation^56^. The different density of the two fibres is due to the differences in size and flexibility of the fibres. In addition, the formed FKFEFKFE nanofibre may not be bound to the liposomes after the propulsion, resulting in a reduction of the propulsive force, as suggested in a review^42^. A possible driving force for the propulsion of **1**-PS and **2**-PS is the Marangoni effect caused by the surface tension gradient between the nanofibre-forming side and the other side of a motor. The surface tension gradient caused by nanofibre growth might be small, which could explain the slow movement of our motors.

**Table 1:** Summary of the tracking trajectories. The diffusion coefficients were calculated from the fitted lines of Fig. 6. The velocities were calculated from the moving distances of liposomes from their initial positions during 10–60 min of measurement, as shown in Fig. 5f. The data represent average ± standard error of mean (N = 20).

Entry	Sample	Light	Diffusion coefficient (μm^2^/min)	Velocity (μm/min)
1	**2**-PS	+	1.29 ± 0.18	0.31 ± 0.04
2	**2**-PS	−	0.56 ± 0.06	0.06 ± 0.02
3	**1**-PS	+	0.78 ± 0.10	0.16 ± 0.02
4	**1**-PS	−	0.55 ± 0.06	0.07 ± 0.02
5	dA_20_-PS	+	0.58 ± 0.07	0.05 ± 0.02

To evaluate the effect of asymmetrical modification of conjugate **2** on PS-liposomes, the movement of a **2**-modified homogeneous liposome (**2**-H) was tracked. The propulsion of the **2**-H was induced by light irradiation (Supplementary Figs 15 and 16). However, the moving distance of **2**-H was shorter than that of **2**-PS (Supplementary Fig. 17). A comparison of the MSD plots also showed that the diffusion enhancement of **2**-H by light was not great compared to that of **2**-PS (Supplementary Fig. 18). In fact, the calculated diffusion coefficient of **2**-H (0.87 ± 0.11 μm^2^/min) under light irradiation was smaller than that of **2**-PS (1.29 ± 0.18 μm^2^/min). These results suggest that asymmetrical fibre formation is important for the propulsion of the liposomes.

In conclusion, we developed novel light-driven motors propelled by peptide nanofibre growth on their surfaces, inspired by natural motors that use actin fibre formation for propulsion. The responsiveness of the motors to light was dependent on the rates of the photocleavage reaction and subsequent fibre formation, indicating that the movement of the motors could be tuned by modifying the photocleavage moiety. These results demonstrated the proof of concept for light-driven motors based on the chemical design of the light-induced peptide nanofibre formation. Elucidation of the detailed mechanism of the propulsion, visualization of the nanofibre formation during the movement of the motors, and modulation of our motors for directional motion are under investigation.

## Method

### Equipment and materials

High-performance liquid chromatography (HPLC) was performed using a Shimadzu LC-6AD liquid chromatograph with GL Science Inertsil WP300 C18 columns (4.6 × 250 mm for analysis and 20 × 250 mm for purification). Matrix-assisted laser desorption/ionisation time-of-flight (MALDI-TOF) mass spectra were taken using a Bruker Daltonics Autoflex TII with α-cyano-4-hydroxycinnamic acid as matrix for CZFKFEFKFE and 3-hydroxypicolinic acid as matrix for dA_20_-maleimide and conjugate **2**. UV-vis spectra were obtained using a Jasco V-630. Transmission electron microscope (TEM) was measured using a Jeol JEM 1400 Plus with a grid (C-SMART Hydrophilic TEM grid, ALLIANCE Biosystems Inc.). Confocal laser scanning microscopy (CLSM) measurement was conducted using a FluoView FV10i (Olympus). Giant liposomes were imaged under an inverted microscope (Eclipse TS100, Nikon) equipped with a digital camera (MC120 HD, Leica). UV irradiation was performed using an LED lump, ULEDN 102-CT (365 nm, 4 W/cm^2^, NS Lighting Co., Ltd.). The reagents were purchased from Watanabe Chemical Ind., Ltd., Tokyo Chemical Industry Co., Dojindo Laboratories Co., Ltd., Avanti Polar Lipids Co. and Wako Pure Chemical Industries. Chemically modified DNAs, dA_20_-NH_2_ (20 mer of deoxyadenosine monophosphate having an amino group at the 5′ end, Supplementary Fig. 1a) and dT_20_-biotin (20 mer of thymidine monophosphate having a biotin group at the 5′ end, Supplementary Fig. 1b) were purchased from Gene Design Inc. All the chemicals were used without further purification.

### Synthesis of conjugates 1 and 2

Conjugate **1** was synthesized according to our previous methods^49^. Conjugate **2** was synthesized by the same methods (Supplementary Scheme 1).

Synthesis of photoresponsive peptide moiety CZFKFEFKFE: CZFKFEFKFE (Z is D-3-amino-3-(2-nitrophenyl)-propionic acid) was synthesized by 9-fluorenylmethyloxycarbonyl (Fmoc) solid phase peptide synthesis. The amino group of each amino acid was protected with Fmoc group. The mercapto, amino, and carboxy groups of C, K, and E were protected with trityl, *tert*-butoxycarbonyl, and *tert*-butyl groups, respectively. Fmoc-D-β-Phe(2-NO_2_)-OH (Watanabe Chemical Ind., Ltd.) was used for Z. To the resin having free amino groups as reaction sites (Fmoc-Glu(OtBu)-Alko PEG Resin, 0.25 mmol/g, 224 mg) were added Fmoc amino acid (4 equiv. for C, Z, K, E and 8 equiv. for F), 1-[(1-(Cyano-2-ethoxy-2-oxoethylideneaminooxy)-dimethylamino-morpholinomethylene)] methanaminium hexafluorophosphate (COMU, 4 equiv. for C, Z, K, E and 8 equiv. for F), and diisopropylethylamine (DIPEA, 4 equiv. for C, Z, K, E and 8 equiv. for F) in *N*-methylpyrrolidone (NMP). Each condensation reaction was performed at room temperature for 2 h. Removal of Fmoc groups from the resin was performed using 40% and 20% piperidine *N*,*N*-dimethylformamide (DMF) solution. Both introduction of Fmoc amino acids and removal of Fmoc groups were checked by a 2,4,6-trinitrobenzene sulfonic acid (TNBS) test kit (Tokyo Chemical Industry Co., Ltd.) after washing the resin with NMP (2 mL × 5). Cleavage of the 10-mer peptide and deprotection at side chains were performed with a cleavage cocktail (trifluoroacetic acid (TFA)/thioanisole/water/ethanedithiol/triisopropylsilane = 81.5/5/5/1/1, v/v/v/v/v). The mixture was stirred at room temperature for 3 h. After filtration, the solution was evaporated, followed by trituration with tert-butylmethylether (25 mL × 3) to give the product in a 29% yield (crude) as white solid. Purification was performed by RP-HPLC with water/acetonitrile (both containing 0.1% TFA, 75/25 to 40/60, v/v for 75 min, linear gradient, 10 mL/min, detected at 220 nm) to give the purified product. MALDI-TOF-MS: *m/z* found: 1419 ([M + H]^+^), calcd. 1418.

#### Synthesis of dA_20_-maleimide

To a 1 mM aqueous solution of dA_20_-NH_2_ (40 nmol, 40 μL) were added *N*-(4-maleimidobutyryloxy)-sulfosuccinimide sodium salt (Sulfo-GMBS, 120 equiv., 1.8 mg, 4.8 μmol) and 0.1 M NaHCO_3_ aqueous solution (120 μL). The mixture was incubated at 25 °C for 4 h. The reaction mixture was dialyzed using a dialysis membrane (Spectra/por7, cutoff Mw: 1,000, Spectrum Laboratories, Inc.) with water for 20 h to remove the excess Sulfo-GMBS. MALDI-TOF-MS: *m/z* found: 6547 ([M + H]^+^), calcd. 6547.

#### Synthesis of conjugate **2**

CZFKFEFKFE (0.43 mg, 300 nmol) in acetonitrile (500 μL) was added to 0.2 M sodium phosphate buffer pH 7.0 (520 μL). Then 100 mM Tris(2-carboxyethyl)phosphine hydrochloride (TCEP-HCl) aqueous solution (30 μL) was added to the solution. The dA_20_-maleimide solution (40 nmol, 450 μL) was added and the mixture was incubated at 40 °C for 48 h. Purification was performed by RP-HPLC with 0.1 M ammonium formate aqueous solution/acetonitrile (95/5 to 0/100, v/v for 95 min, linear gradient, 10 mL/min, detected at 260 nm) to give the purified product. MALDI-TOF-MS: *m/z* found: 7964 ([M + H]^+^), calcd. 7964 (Supplementary Fig. 2).

### Preparation of giant liposomes

Phase-separated giant liposome (PS-liposome, Supplementary Fig. 11) was prepared by a natural swelling method using D-glucose^57^. DPPC/DOPC/DOPE-biotin/Chol with the molar ratios of 5:4:1:4.5 were mixed with D-glucose (1 equiv. to the lipids) in chloroform/methanol (2/1, v/v). The solution in a glass test tube (i.d. ~1 cm) was dried in vacuo for 1–2 days. The dried film was then hydrated at 50 °C with water (500 μL) for 1 h. The final concentration of lipids was 0.1 mM. Homogeneous liposome (H-liposome) was prepared by the same method using the lipids consisting of DOPC/DOPE-biotin/Chol with the molar ratios of 9:1:4.5. The liposomes were stored under a nitrogen atmosphere at 4 °C and used within a week.

### Observation of movement of liposomes

To conjugates **1**, **2** or dA_20_-NH_2_ (15 μM, 1 μL) were sequentially added dT_20_-biotin (10 μM, 1 μL), streptavidin (10 μM, 1 μL), D-glucose (400 μM, 1 μL), and the liposomes (1 μL, containing 10 μM DOPE-biotin). The setting of the observation system is shown in Supplementary Fig. 13. To close off the sample from its surroundings, a square, a double-sided piece of tape (0.05 mm-thick, ESCO, Japan) with the center removed was adhered onto a cover glass (24 mm × 60 mm, Matsunami, Japan) to create a well (*ca*. 12 mm × 12 mm). The liposome solution (2 μL) was added to the well, then a cover glass (18 mm × 18 mm, Matsunami, Japan) was adhered to the top of the tape. After standing for 2 h, the images were recorded as a video for 60 min. For light-irradiation experiment, the samples were kept under dark for 10 min, irradiated with UV light (365 nm, 4 W cm^−2^) for 10 min with 4 cm distance from the center of the well, then again kept under dark for 40 min. Because the distance between the light source and sample was little longer (4 cm) compared to *in vitro* uncaging experiments (3 cm), we irradiated the UV light for 10 min for complete uncaging. The trajectory of the liposomes was tracked by analysing the images by a software ImageJ (*n* ≥ 10).

## Electronic supplementary material

Supplementary Information

Supplementary Video 1
