# Supplementary material for: Light-induced propulsion of a giant liposome driven by peptide nanofibre growth
Source: Sci Rep. 2018 Apr 19;8:6243. doi: 10.1038/s41598-018-24675-7 (PMC5908854; doi:10.1038/s41598-018-24675-7)
Supplement: Supplementary file 1 — Supplementary Information [file 41598_2018_24675_MOESM1_ESM.docx]

Supplementary Information

**Light-induced propulsion of a giant liposome driven by peptide nanofibre growth**

Hiroshi Inaba^1^*, Akihito Uemura^1^, Kazushi Morishita^1^, Taiki Kohiki^2^, Akira Shigenaga^2^, Akira Otaka^2^ and Kazunori Matsuura^1^*

^1^Graduate School of Engineering, Tottori University, 4-101 Koyama-Minami, Tottori 680-8552, Japan.

^2^Institute of Biomedical Sciences and Graduate School of Pharmaceutical Sciences, Tokushima University, Shomachi, Tokushima 770-8505, Japan.

**Kinetics of photocleavage reactions of conjugates 1 and 2.** The photocleavage reactions of conjugates **1** and **2** were monitored using RP-HPLC, and the processing products were characterised by MALDI-TOF-MS. The production of DNA moiety (Intermediate and dA_20_(1) for conjugate **1** and dA_20_(2) for conjugate **2**) were analysed by detection at 260 nm (Supplementary Scheme 2 and 3). Analytical RP-HPLC was performed with 0.1 M ammonium formate/acetonitrile (95/5 to 0/100, v/v for 95 min, linear gradient, 1 mL/min).

For the detection of the intermediate of conjugate **1**, UV light (365 nm, 4 W cm^–2^) was irradiated into 5.6 μM conjugate **1** in water/acetonitrile (9/1, v/v, 10 μL) with 3 cm distance from the sample for 0-30 sec, then the samples were immediately analysed by RP-HPLC (Supplementary Fig. 3). MALDI-TOF-MS of the intermediate isolated by RP-HPLC is shown in Supplementary Fig. 4. To evaluate the kinetics of intramolecular cyclisation of the intermediate, UV light was irradiated to 5.6 μM conjugate **1** in water/acetonitrile (9/1, v/v, 10 μL) for 30 sec, then the sample was incubated at 25°C for 0-130 min and analysed by RP-HPLC (Supplementary Fig. 5). MALDI-TOF-MS of the dA_20_ moiety (dA_20_(**1**)) isolated by RP-HPLC is shown in Supplementary Fig. 6. For the analysis of the photocleavage reaction of conjugate **2**, UV light (365 nm, 4 W cm^–2^) was irradiated into 5.6 μM conjugate **2** in water/acetonitrile (9/1, v/v, 10 μL) with 3 cm distance from the sample for 0-120 sec, then the samples were immediately analysed by RP-HPLC (Supplementary Fig. 7). MALDI-TOF-MS of the dA_20_ moiety (dA_20_(**2**)) isolated by RP-HPLC is shown in Supplementary Fig. 8.

**Turbidity measurement.** Turbidity experiments were performed with 5.6 μM conjugate **1** or **2** in water/acetonitrile (9/1, v/v, 100 μL) at 25°C. Optical density at 400 nm was monitored for 60 min at 30 sec intervals. The absorbance was measured for 5 min and thereafter UV light (365 nm, 4 W cm^–2^) was irradiated with 3 cm distance from the sample in a cuvette for 3 min.

**TEM measurement.** Conjugate **2** (Supplementary Fig. 10): UV light (365 nm, 4 W cm^–2^) was irradiated to 5.6 μM of conjugate **2** in water/acetonitrile (9/1, v/v, 5 μL) for 3 min. The solution was incubated at 25°C for 30 min and put on a TEM grid, allowed to stand for 1 min, and then removed. The grid was exposed to 2% phosphotungstic acid aqueous solution (5 L), which was allowed to stand for 1 min, and then removed. The resulting grid was dried in vacuo and used. Before UV irradiation, conjugate **2** was only spherical structures with a size of ca. 10 nm due to the dispersion (Supplementary Fig. 10a). After UV irradiation, fibrous assemblies 20–30 nm in width were observed (Supplementary Fig. 10b). These results indicate that the peptide moiety was released by UV irradiation to self-assemble into nanofibres.

Liposomes (Supplementary Fig. 12): UV light (365 nm, 4 W cm^–2^) was irradiated to **1**-PS (2 μL) for 3 min. The solution was put on a TEM grid, allowed to stand for 1 min, and then removed. The grid was exposed to 2% phosphotungstic acid aqueous solution (2 L), which was allowed to stand for 1 min, and then removed. The resulting grid was dried in vacuo and used.

**Confocal laser scanning microscopy (CLSM).** To the giant liposomes (2 μL, containing 10 μM DOPE-biotin) were added FITC-streptavidin (10 μM, 1 μL) and Nile red (8 μM, 0.65 μL). After incubation at room temperature for 1 h, the liposomes were imaged. FITC-streptavidin was excited with 495 nm and observed through a 519 nm emission band-pass filter (Green). Nile red was excited with 559 nm and observed through a 570-620 nm emission band-pass filter (Red).

**Supplementary Figure 1.** Structures of (a) dA_20_-NH_2_ and (b) dT_20_-biotin.

**Supplementary Scheme 1.** Synthesis of conjugate **2**.

**Supplementary Figure 2.** MALDI-TOF-MS of conjugate **2**.

**Supplementary Scheme 2.** Photocleavage reaction of conjugate **1**.

**Supplementary Figure 3.** (a) HPLC charts of conjugate **1** upon different light irradiation time. (b) Peak area of conjugate **1** obtained from (a). (c) First-order kinetic treatment of (b). Peak area of conjugate is shown as [**1**].

**Supplementary Figure 4.** MALDI-TOF-MS of the intermediate isolated by HPLC in Supplementary Fig. 3a.

**Supplementary Figure 5.** (a) HPLC charts of conjugate **1** upon 30 sec light irradiation and subsequent different incubation time at 25°C. (b) Peak area of Intermediate obtained from (a). (c) First-order kinetic treatment of (b). Peak area of Intermediate is shown as [Intermediate].

**Supplementary Figure 6.** MALDI-TOF-MS of dA_20_(1) isolated by HPLC in Supplementary Fig. 5a.

**Supplementary Scheme 3**. Photocleavage reaction of conjugate **2**.

**Supplementary Figure 7.** (a) HPLC charts of conjugate **2** upon different light irradiation time. (b) Peak area of dA_20_(2) obtained from (a). (c) First-order kinetic treatment of (b). Peak area of dA_20_ moiety is shown as [dA_20_(2)].

**Supplementary Figure 8.** MALDI-TOF-MS of dA_20_(2) isolated by HPLC in Supplementary Fig. 7a.

**Supplementary Figure 9.** Time course of turbidity change (optical density at 400 nm) of conjugates **1** and **2** (5.6 μM) in water/acetonitrile (9/1, v/v) at 25°C. UV irradiation was continuously conducted for 3 min after 5 min incubation under darkness.

**Supplementary Figure 10.** TEM images of conjugate **2** observed (a) before and (b) after UV light irradiation. The samples were stained with 2% aqueous solution of sodium phosphotungstate.

**Supplementary Figure 11.** Asymmetrically modified conjugate **1** or **2** on a PS-liposome.

**Supplementary Figure 12.** TEM images of **1**-PS (a) without and (b) with UV light irradiation. The samples were stained with 2% aqueous solution of sodium phosphotungstate.

**Supplementary Table 1.** Diameter of liposomes used for the microscope observation. The data represent average ± standard deviation.

| **Entry** | **Sample** | **Light** | **Diameter (μm)** |
| --- | --- | --- | --- |
| 1 | **2**-PS | + | 14.7 ± 3.6 |
| 2 | **2**-PS | – | 16.2 ± 3.2 |
| 3 | **1**-PS | + | 14.9 ± 4.3 |
| 4 | **1**-PS | – | 15.6 ± 3.8 |
| 5 | dA_20_-PS | + | 13.6 ± 3.2 |
| 6 | **2**-H | + | 13.5 ± 2.8 |
| 7 | **2**-H | – | 14.9 ± 5.5 |

**Supplementary Figure 13.** Schematic illustration of an observation system for measurement of movement of giant liposomes under light irradiation.

**Supplementary Figure 14.** Tracking trajectories of **1**-PS during 60 min recording (a) with and (b) without UV light, and (c) dA_20_-PS with UV light. UV light was irradiated between the 10 to 20 min recording. Each color represents different liposome (*N* =20).

**Supplementary Figure 15.** Tracking trajectories of **2**-H during 60 min recording (a) with and (b) without UV light. UV light was irradiated between the 10-20 min recording. Each color represents different liposome (*N* =20).

**Supplementary Figure 16.** Time dependence of the distance of **2**-H from the initial position under UV light irradiation (orange zones) and non-irradiation (white zones), analysed from the trajectory tracking. Each color represents different liposome (*N* =20).

**Supplementary Figure 17.** Time dependence of the average distance of **2**-PS (red) and **2**-H (black) from the original position under UV light irradiation (orange zones) and non-irradiation (white zones), analysed from the trajectory tracking. Solid lines and dashed lines indicate the light-irradiated and non-irradiated samples, respectively. The plots of **2**-PS are same as Fig. 5f.

**Supplementary Figure 18.** Fitting plots of mean square displacement (MSD) versus time interval (Δ*t*), analysed from the trajectory tracking of 20 liposomes during 10–60 min of measurement. The plots of **2**-PS are same as Fig. 6.
